# Advances in engineered organoid models of skin for biomedical research

**DOI:** 10.1093/burnst/tkaf016

**Published:** 2025-02-23

**Authors:** Dongao Zeng, Shikai Li, Fangzhou Du, Yuchen Xia, Jingzhong Zhang, Shuang Yu, Jianhua Qin

**Affiliations:** Suzhou Institute of Biomedical Engineering and Technology, Chinese Academy of Sciences, No. 88, Keling Road, Suzhou New District, Suzhou, Jiangsu Province 215163, China; School of Biomedical Engineering (Suzhou), Division of Life Sciences and Medicine, University of Science and Technology of China, No.96 Jinzhai Road, Hefei, Anhui Province 230026, China; Suzhou Institute of Biomedical Engineering and Technology, Chinese Academy of Sciences, No. 88, Keling Road, Suzhou New District, Suzhou, Jiangsu Province 215163, China; School of Biomedical Engineering (Suzhou), Division of Life Sciences and Medicine, University of Science and Technology of China, No.96 Jinzhai Road, Hefei, Anhui Province 230026, China; Suzhou Institute of Biomedical Engineering and Technology, Chinese Academy of Sciences, No. 88, Keling Road, Suzhou New District, Suzhou, Jiangsu Province 215163, China; Suzhou Institute of Biomedical Engineering and Technology, Chinese Academy of Sciences, No. 88, Keling Road, Suzhou New District, Suzhou, Jiangsu Province 215163, China; School of Biomedical Engineering (Suzhou), Division of Life Sciences and Medicine, University of Science and Technology of China, No.96 Jinzhai Road, Hefei, Anhui Province 230026, China; Suzhou Institute of Biomedical Engineering and Technology, Chinese Academy of Sciences, No. 88, Keling Road, Suzhou New District, Suzhou, Jiangsu Province 215163, China; School of Biomedical Engineering (Suzhou), Division of Life Sciences and Medicine, University of Science and Technology of China, No.96 Jinzhai Road, Hefei, Anhui Province 230026, China; Xuzhou Medical University, No. 209 Tongshan Road, Xuzhou, Jiangsu Province 221004, China; Suzhou Institute of Biomedical Engineering and Technology, Chinese Academy of Sciences, No. 88, Keling Road, Suzhou New District, Suzhou, Jiangsu Province 215163, China; School of Biomedical Engineering (Suzhou), Division of Life Sciences and Medicine, University of Science and Technology of China, No.96 Jinzhai Road, Hefei, Anhui Province 230026, China; Xuzhou Medical University, No. 209 Tongshan Road, Xuzhou, Jiangsu Province 221004, China; School of Biomedical Engineering (Suzhou), Division of Life Sciences and Medicine, University of Science and Technology of China, No.96 Jinzhai Road, Hefei, Anhui Province 230026, China; Division of Biotechnology, Dalian Institute of Chemical Physics, Chinese Academy of Sciences, No. 457 Zhongshan Road, Dalian, Liaoning Province 116023, China

**Keywords:** Organoid models of skin, Skin development, Skin appendages, Stem cells, Bioengineering methods, 3D scaffolds, Signaling pathways

## Abstract

In recent years, significant progress has been made in the development of organoids, which offer promising opportunities for developmental and translational research. With advances in cell biology and bioengineering techniques, skin models are evolving from conventional multilayered structures to appendage-bearing spheroids or 3D biomimetic models. This comprehensive review aims to provide an in-depth understanding of organoid models of the skin, covering topics such as skin development, construction strategies and key elements, types of organoid models, biomedical applications, and challenges. Embryonic skin development is briefly introduced to provide a foundational understanding of construction principles. Current engineering strategies are outlined, highlighting key elements such as cell sources, bioengineering techniques, 3D scaffolds, and crucial signaling pathways. Furthermore, recent advances in generating organoids with structural and functional parallels to native skin are meticulously summarized. These developments facilitate the utilization of organoids in diverse applications, such as modeling skin disorders, developing regenerative solutions, and understanding skin development. Finally, the challenges and prospects in the field are discussed. The integration of state-of-the-art bioengineering techniques with a deep understanding of skin biology is promoting the production and biomedical application of these organoid models.

## Graphical Abstract

**Graphical Abstract:**
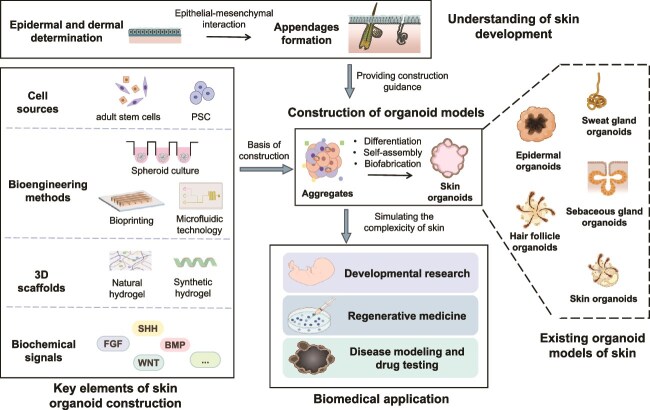

Highlights

- Dissection of construction strategies by integrating insights from skin developmental biology.
- Comprehensive overview of key elements, including cell sources, bioengineering techniques, 3D scaffolds, and crucial signaling pathways.
- Summary of recent progress in engineered skin organoids and their diverse applications in research and medicine.

## Background

The skin, as the body's largest organ, is responsible for numerous essential physiological functions, including barrier protection, homeostasis, endocrine regulation, and sensation. Structurally, the skin can be divided into three distinct layers: the epidermis, dermis, and subcutaneous tissue [1, 2]. Skin appendages such as hair follicles (HFs), sebaceous glands (SeGs), and sweat glands (SGs) are embedded within the skin and perform various functions, including hair growth, thermoregulation, secretion, and metabolism [1, 2]. Additionally, the skin is a complex organ that contains various cell types from multiple embryonic origins. The epidermis, which is of ectodermal origin, is a stratified squamous epithelium containing epidermal stem cells (EpSCs), keratinocytes (KCs), melanocytes, Langerhans cells, Meckel cells, etc. [3] The dermis, which is derived primarily from a mesodermal origin, is composed mainly of fibroblasts and extracellular matrix (ECM). Appendages such as HFs, SeGs, and SGs are located in the dermis and extend into the epidermis [4]. Subcutaneous tissue contains fat and connective tissue, and it provides nutrients and support to the skin [1, 2]. Because of the complexity of the structure and function of the skin, establishing an *in vitro* model that accurately mimics native skin has proven to be challenging.

The organoid technique provides new approaches to recapitulate the complexity of skin. An organoid is a 3D multicellular structure derived from stem cells *in vitro* that mimics the organ or disease they represent. Organoids form mainly through self-organization and can better recapitulate the specific structure and function of the corresponding *in vivo* tissue [5, 6]. The earliest documented 3D skin model can be traced back to the 1970s, when Rheinwald and Green developed a groundbreaking technique to cultivate KCs at the air–liquid interface (ALI), leading to the differentiation and formation of stratified layers resembling epidermal tissue [7, 8]. This pioneering work laid the foundation for the development of 3D skin models, with the ALI model emerging as the dominant skin construct and undergoing extensive use in subsequent decades. The ALI model successfully achieved barrier properties and phenotypic differentiation; however, the inclusion of appendages such as HFs and SGs has yet to be realized [9]. With advances in bioengineering techniques, biomaterials and the understanding of skin development, new organoid models integrating skin appendages have been established. The donor cells include not only the skin cells but also adult stem cells in other body parts, as well as embryonic stem cells (ESCs) and induced pluripotent stem cells (iPSCs). The bioengineering methods have significantly diversified to include spheroid culture, 3D bioprinting, and microfluidic techniques, and the utilization of 3D scaffolds has been enriched by the introduction of a wider array of natural and synthetic materials. To date, 3D skin constructs such as epidermal organoids [10–12], HF organoids [13], SeG organoids [1, 4] and SG organoids [14–20], as well as appendage-bearing multilayered skin organoids [21–28], have been established. In this review, these constructs are summarized as organoid models of skin, as they are developed from stem cells, contain multiple cell types, and exhibit distinct morphological, and functional characteristics of skin.

In this review, we provide an overview of the advances in creating organoid models of skin. We aim to provide a comprehensive understanding of the development of skin-based organoids, covering topics of *in vivo* skin development, construction strategies and key elements, various types of skin-based organoid models, biomedical applications, challenges, etc. Given extensive previous reviews of ALI models, we focus on recent advances in organoid models of skin developed through self-assembly or the use of cutting–edge bioengineering techniques. With ongoing advances in bioengineering technologies and an enhanced understanding of skin development, organoid models of skin hold broad prospects for application in the biomedical field.

## Review

### *In vivo* skin development

**Figure 1:**
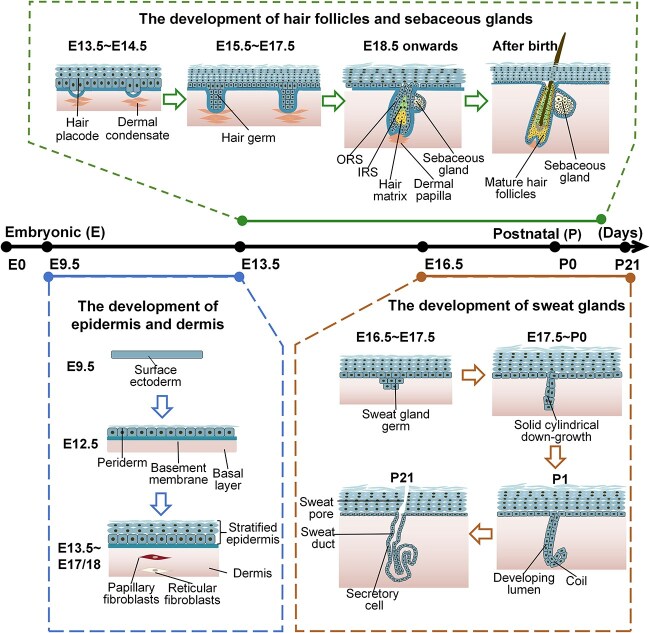
Schematic diagram of skin development and appendage formation. Starting at embryonic day 9.5 (E9.5), the single-layered epidermis undergoes proliferation and stratification, leading to the formation of a stratified epidermis (E13.5–E17/18). Close interactions between hair placodes and DCs (E13.5–E14.5) lead to the gradual formation of hair germs (E15.5–E17.5). These hair germs undergo differentiation, giving rise to HFs and subsequently SeGs, which appear at approximately E18.5 and become functional after birth. SG germ begins to form just before birth (E16.5–17.5). Progenitor cells originating from the epidermal basal layer invaginate and extend deeply into the dermis between E17.5 and postnatal day 0 (P0), subsequently differentiating into secretory coils by P1. The glands achieve full functionality by P21. *ORS* outer root sheath, *IRS* inner root sheath

Understanding *in vivo* skin development is crucial for guiding the construction of skin models. Mammalian skin development is a multistage process that includes the formation of the epidermis and dermis, along with the growth of skin appendages, including HFs, SeGs, and SGs (Figure 1) [13, 29]. In mice, the development of the epidermis begins from the surface ectoderm at embryonic day (E) 9.5 [30]. The ectoderm receives signals from the mesoderm, which inhibits neural differentiation and promotes epidermal fate determination [31–33]. Prior to the key developmental milestone at E12.5, the majority of basal progenitor cells in murine skin divide symmetrically along the axis of the basement membrane. From E13.5 onward, more basal progenitor cells shift their division axis in a perpendicular orientation, undergoing asymmetric division and contributing to the formation of a stratified epidermis [34, 35]. During the initial stages of stratification, a temporary protective layer called the periderm, consisting of tightly connected squamous cells, covers the epidermis. The periderm is shed at approximately E17–18, indicating the completion of stratification [14, 15, 29].

Unlike the ectodermal origin of the epidermis, the embryonic origins of dermal fibroblasts in mammals are dependent on anatomical location [13, 16, 29]. In the facial and anterior head regions, dermal fibroblasts are derived from cranial neural crest (NC) cells, whereas those in the posterior head region arise from the cephalic mesoderm [17]. Fibroblasts in the dorsal and ventral regions originate from somite dermomyotomes and lateral plate mesoderm, respectively [18–20]. To date, a comprehensive molecular understanding of how these fibroblast populations develop is lacking. The dorsal trunk in mouse embryos has been extensively studied with regard to the development and specification of fibroblasts. After dermal specification, canonical wingless-related integration site (WNT) signaling drives the differentiation of dermal fibroblasts into distinct papillary and reticular subpopulations, which can be distinguished at E16.5 by using lineage tracing methods [21]. Recently, with advances in single-cell sequencing techniques, the diversification of fibroblasts into functional lineages was reported to occur as early as E12.5 [30].

The development of skin appendages depends primarily on reciprocal epithelial and mesenchymal interactions (EMIs). The morphogenetic signals HF formation arise primarily from the dermis. At E13.5–14.5, specific subsets of mesenchymal cells aggregate to form dermal condensates (DCs) that determine HF locations and induce hair placode formation [22, 23]. Crosstalk between DCs and hair placodes drives further maturation of the HF and subsequent hair growth cycles [22, 24]. At approximately E18.5, SeGs become apparent, typically coinciding with the maturation of HFs [14, 25]. At E16.5–17.5, SGs start to develop as epithelial invaginations. These invaginations undergo further maturation, forming elongated ducts that penetrate into the dermis and become fully functional and mature by postnatal day 21 [26, 27].

**Figure 2:**
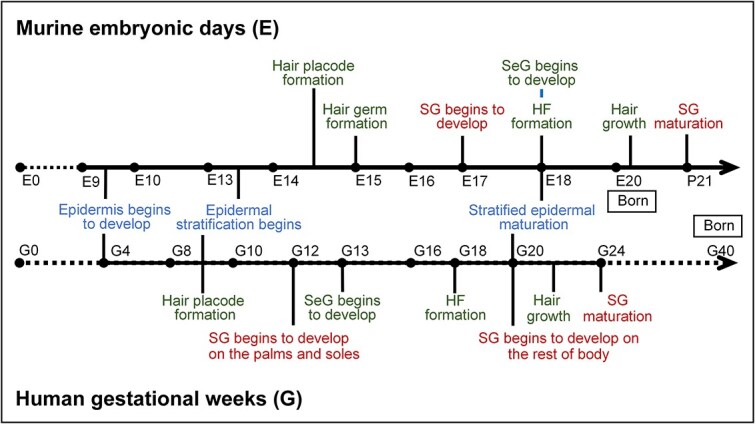
The timelines of murine *versus* human skin development. In mice, stratification of the epidermis begins at approximately embryonic day (E) 9.5 and concludes by E18, whereas in humans, epidermal stratification starts as early as 4 weeks of gestation (G4) and is completed by G20. Additionally, mice begin developing HFs at E14.5 and achieve fully mature HFs and hair growth after birth, whereas human HF development begins at G18, with hair growth beginning at G22. SG development in mice begins just prior to birth (E18), whereas in humans, SGs reach functional maturity by G24. *SeG* sebaceous gland

The development of human skin shares broad similarities with that of rodent skin. The human epithelium also originates from the embryonic ectoderm and undergoes processes of proliferation, differentiation, stratification, and homeostasis. The origin of the dermis is diverse, and the reciprocal interaction between the epidermis and dermis determines the development of skin appendages [36]. However, notable differences exist between these two species (Figure 2). For example, epidermal stratification in mice occurs during mid- to late-embryonic development, whereas in humans, it takes place from early to mid-gestation. In terms of skin appendages, the first hair growth wave in mice generally occurs postnatally, whereas in humans, mature HFs are already present at 22 weeks of gestation. Similarly, SGs in mice mature after birth, whereas in humans, they undergo differentiation and maturation by 24 weeks of gestation. Additionally, there are anatomical and distribution differences between murine and human skin [28, 37], such as differences in the thickness of the epidermis and dermis, as well as the locations of HFs and SGs [28]. The distribution of HFs is sparse in humans and dense in mice. Human skin has both eccrine and apocrine SGs, with the former being widely distributed across the body and the latter being confined to very hairy body regions. In contrast, mice possess only eccrine SGs, which are exclusively located in the paw pads ([26]). Consequently, skin organoids derived from human tissue can more precisely mimic skin development and the onset and progression of skin diseases.

### Construction strategies and key elements to develop organoid models of skin

**Figure 3:**
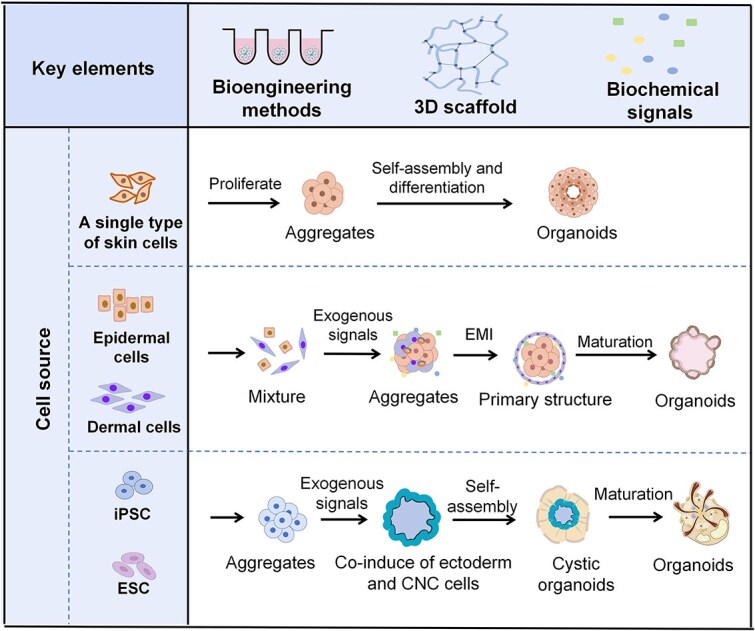
Schematic illustration of skin-based organoid generation. Several key elements are required to generate organoid models of skin. These include the following: (i) Suitable cell sources include single or multiple types of primary skin cells derived from biopsies, as well as pluripotent embryonic stem cells (ESCs) and iPSCs. (ii) selection of proper bioengineering techniques to support the construction of organoids. (iii) 3D scaffolds provide support for the attachment and organization of cells. (iv) Manipulation of proper biochemical signals. Growth factors and supplements were added to regulate specific signaling pathways, mimicking the environment needed for proper cell behavior and development. Organoid models have been constructed by integrating these key elements via different strategies. The first approach involves aggregating a single type of skin stem cell to form organoids, such as epidermal organoids or SeG organoids. The second strategy employs epidermal and dermal-derived cells, following the principle of EMIs to self-organize into organoids, such as HF organoids. The third strategy involves the directed differentiation of multipotent ESCs or iPSCs into both epithelial and mesenchymal cells, which subsequently develop into skin organoids through self-assembly. CNC, cranial neural crest. Created with BioRender.com

The precise understanding of embryonic skin development provides the foundation for accurately recapitulating the complexity and functionality of skin in organoid models. By offering stem cells a suitable microenvironment that simulates *in vivo* skin development, scientists have developed diverse skin-based organoids that accurately recapitulate the intricate structure and function of the skin (Figure 3). Three main strategies have been employed to develop organoid models of skin *in vitro*. The first strategy involves the use of a single type of skin cell to generate organoids. Stem cells from the epidermis or dermis aggregate *in vitro* and develop into organoids with the aid of various biomaterial scaffolds and signaling molecules [10–12, 38–41]. Because of the crucial role of EMIs in skin appendage development, many researchers have adopted another approach to develop organoid models of the skin. Cells derived from both the epidermis and dermis are isolated and induced to self-organize *in vitro*, leading to the formation of various skin appendage organoids, particularly HFs [42–46]. The third strategy involves the directed differentiation of ESCs or iPSCs into both epithelial and mesenchymal cells, allowing the two distinct cell populations to organize *in vitro*, replicating the process observed in embryonic skin development. By guiding the differentiation of PSCs into the starting population of skin progenitors, researchers have generated skin organoids that mimic the skin developmental process, including appendage induction [47–50]. These strategies involve the interaction of four key elements: cell sources, bioengineering methods, 3D scaffolds, and biological signals. These elements complement each other and collectively establish the foundation for the creation of organoid models.

#### Cell sources

Stem cells, which include adult stem or progenitor cells and PSCs, are essential for organoid formation and growth [6, 51]. Adult stem or progenitor cells are typically found in specific areas of the body and have limited self-renewal and differentiation ability [52–54]. The creation of skin-based organoids involves the use of cells from various layers of the skin, including EpSCs [11, 55], KCs [10, 12, 56], dermal fibroblasts [46, 57], HF stem cells [58], dermal papilla cells (DPCs) [59–63], and SG cells [40, 41]. These organoids can also be produced from adult stem cells from different body parts, such as mesenchymal cells from the bone marrow [64, 65] and adipose tissue [66].

Additionally, skin organoids can be generated from PSCs through stepwise differentiation and self-assembly [47, 48, 50, 67–69]. PSCs can be ESCs derived from embryos or iPSCs [70] generated through induced dedifferentiation from somatic cells via cell reprogramming. PSCs can undergo indefinite self-renewal and give rise to all cell types in the body, but this versatility also increases the risk of off-target induction [48, 71].

#### Bioengineering methods

**Figure 4:**
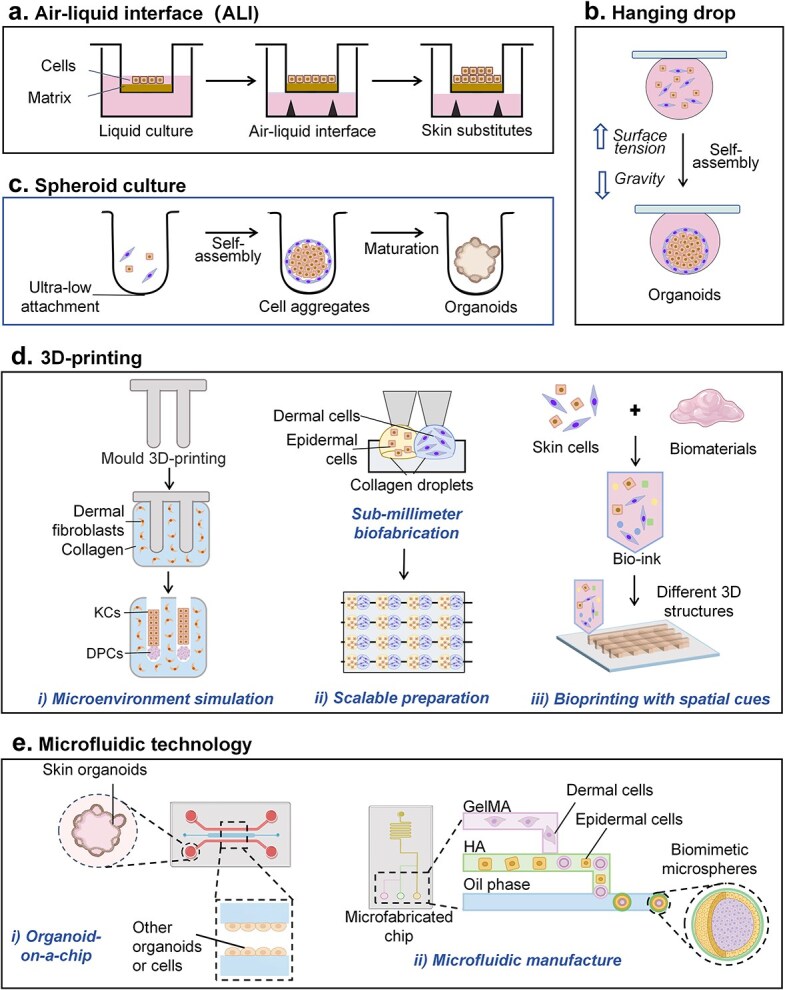
Different bioengineering methods for constructing organoid models of skin. (**a**) In the classical ALI model, cells are plated on a membrane and allowed to reach confluence in culture medium. They are then exposed to air, prompting their development into stratified skin substitutes. (**b**) The hanging-drop method involves the aggregation and maturation of cells at the liquid–air interface of hanging droplets. (**c**) In spheroid culture, stem cells are plated in biomaterials and cultured in ULA plates to self-assemble into spherical aggregates. (**d**) Organoid construction via 3D printing. (i) To simulate the microenvironment of HFs, special 3D molds are printed, followed by sequential assembly of extramold fibroblasts and collagen, as well as intramold DPCs and keratinocytes. (ii) To prepare HF microgels, two tiny adjacent collagen droplets containing dermal and epidermal cells are bioprinted on a matrix, and subsequent spontaneous contraction significantly enhances EMIs during HF formation. These bioprinting techniques can be scaled up using an automated spotter. (iii) Computer-assisted 3D bioprinting techniques facilitate the automated and simultaneous spatial arrangement of cells and biomaterials; for example, these strategies have been used in the construction of SG organoids. (**e**) Microfluidic technology combined with skin models. (i) The microfluidic chip comprises a network of grooves or microchannels etched into various substrates, which allows for the designed assembly of skin models and other types of organoids or cells. Microfluidic technology is a computer-aided technique that provides precise control and analysis of physicochemical reactions inside a chip. (ii) Microfluidic technology is employed for the one-step generation of biomimetic microspheres. These double-layer microspheres entrap double-layered cells and bioactive molecules, which act as a bionic organoid model to induce hair formation

In general, there are several main methods for constructing organoids (Figure 4), including the ALI method, hanging–drop method, spherical aggregation method, 3D printing technique, and microfluidic method. As described above, the ALI method is a well-established technique for constructing epithelial models, such as skin and airway models [72–74]. In this method, apical, and basolateral compartments are separated by a permeable membrane. After being seeded on the membrane, the cells were grown to confluence in the culture medium. The medium on the apical side was subsequently removed to expose the cells to air, initiating the process of differentiation. This epidermal analog included stratified epithelial layers expressing structural proteins such as keratin 10 and tight junction proteins such as claudin and occludin [75]. Alternatively, a full-thickness human skin equivalent can be prepared by combining the epidermis with fibroblast-seeded collagen or a fibrin matrix [46, 76, 77]. Although ALI skin equivalents have been widely used to evaluate damage to the skin barrier, it should be noted that such skin-like structures lack skin appendages and have a limited lifespan (~2 weeks) [9, 78].

The hanging drop method is a special form of the ALI technique that depends on cell aggregation at the liquid–air interface to form spheroids [62, 79]. Some disadvantages of this method include the inability to hold droplets >50 μl and to change the medium without disturbing the spheroids [62, 80].

One of the most widely adopted techniques in organoid research was developed by the Hans Clevers team. In this method, stem cells are plated in Matrigel or another ECM matrix and cultured in low-adhesion plates to self-assemble into spherical aggregates at the bottom [81]. Most of the organoid models of skin mentioned in the review were developed by using this method. This ECM scaffold method is rather simple to perform, allows high throughput, and has the potential for the fusion and coculture of multiple organoids.

Recently, 3D printing has gained considerable attention in organoid fabrication. This technique enables the precise and versatile printing of living cells and multiple biomaterials, facilitating the construction of complex structures [76, 82]. For example, researchers have utilized 3D printing technology to create specialized molds resembling human HF structures, enabling physiological 3D cell organization within a suitable microenvironment [44]. Bioprinting also enables the fabrication of tiny objects with high accuracy, allowing scalable automated biomanufacturing [42, 83]. As an example, hair microgels can be prepared by bioprinting two tiny adjacent collagen droplets, one with mesenchymal cells and the other with epithelial cells. During culture, these microgel pairs contract due to cell and collagen traction forces, resulting in >10-fold enrichment in cell density. Scaling up this technique using an automated spotter enables the preparation of large numbers of hair microgels, which is crucial for clinical applications [42]. Moreover, Nanmo *et al.* successfully printed hair microgels onto surgical suture guides, leading to significant improvement in hair-shaft sprouting through the skin when these guide-inserted hair microgels were used [42, 84]. 3D bioprinting strategies also enable the simultaneous spatial patterning of cells and biomaterials. For example, mesenchymal stem cells (MSCs) can be printed in a specific bioink that provides biochemical and architectural cues for the conversion of MSCs into SG cells, ultimately leading to the formation of SG organoids [64, 65].

As organoid technology continues to advance, microfluidic organoid-on-chip technology has been developed on the basis of various organoid construction methods [85]. The reported skin-on-a-chip models integrate an ALI skin model within a microfluidically controlled microenvironment, closely mimicking the mechanical forces and biochemical cues encountered by natural human skin. [9, 86, 87] Such microfluidic skin models have demonstrated significant potential, as they enhance nutrient perfusion, prevent necrosis, and enable the accurate replication of cell–cell contacts, matrix properties, mechanical and biochemical cues and stimuli [9, 87–89]. It is foreseeable that an increasing number of skin or appendage organoids will be coupled with microfluidic techniques to produce powerful models for biomedical research. Microfluidic technology might also be introduced in the generation of bioengineered organoids. In a recent report, microfluidic technology enabled the one-step generation of core–shell biomimetic microspheres. These microspheres consist of double aqueous microdroplets that encapsulate double-layer cells and growth factors, creating a highly mimetic environment for hair regeneration. This innovative technique enables the rapid and scalable preparation of double-layer cell spheres specifically designed for hair regeneration [66].

#### Three-dimensional scaffolds for microenvironment simulation

**Figure 5:**
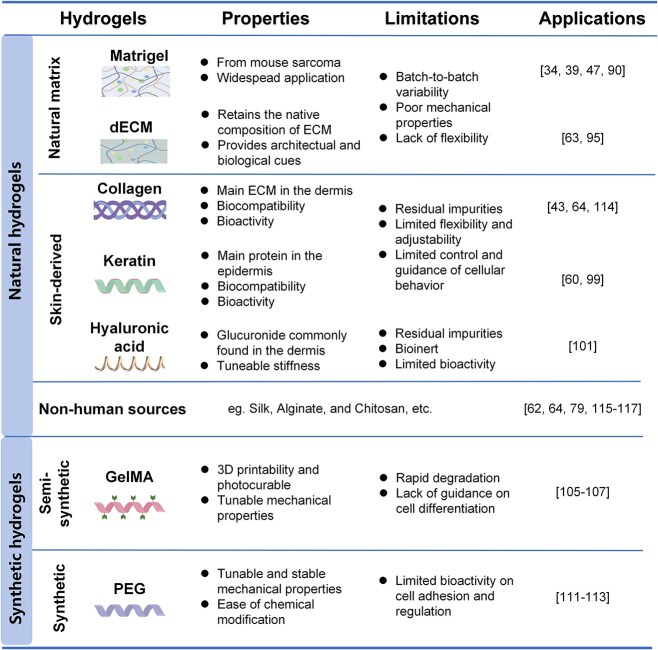
Overview of the hydrogels commonly used in the generation of organoids, especially organoid models of skin

Bioengineering techniques are often supplemented by the utilization of biomaterials to mimic the intricate structure and components of human skin [90]. Hydrogels are widely used to provide 3D scaffolds for organoid construction because of their advantages of biocompatibility, controllability, and delivery capability [91, 92]. Hydrogels include natural hydrogels, such as Matrigel, decellularized ECM (dECM), collagen, keratin, hyaluronic acid (HA), alginate and chitosan, and synthetic hydrogels (Figure 5). [91, 92].

Matrigel, derived primarily from Engelbreth–Holm–Swarm mouse sarcoma, is a solubilized basement membrane extract containing various ECM proteins and growth factors [93]. It has been widely used to culture all types of organoids, including skin organoids [10, 40, 48, 94]. Although Matrigel provides a favorable microenvironment for skin organoid development, its rigidity upon solidification is considerably lower than that of skin tissue [95]. This disparity can potentially affect the determination of the fate of skin stem cells [96]. In addition, its ill-defined composition, batch-to-batch variability and animal-derived nature lead to experimental uncertainty and variable outcomes in organoid protocols [97].

Like Matrigel, dECM is a heterogeneous extract derived from various tissues. The tissue-specific dECM modulates cell morphology and behavior by providing inherent instructive signals that guide tissue morphogenesis [98]. For example, Zhang *et al.* successfully restored the hair-inducing properties of high-generation DPCs by culturing them in dECM from human placental cells [99]. Yao *et al.* used a specific matrix from the mouse plantar region dermis to guide the fate conversion of MSCs to SG cells [64]. In addition, functional chemicals and proteins are frequently used in combination with dECM to increase performance [100].

With the development of organoid culture technology, proteins or polysaccharides from natural tissues with well-defined biochemical compositions and mechanical properties have been employed to construct organoid models of skin [2, 101]. Collagen and keratin are the most abundant ECM proteins in the dermis and epidermis, respectively [102]. For example, Abaci *et al.* used collagen and fibroblasts to mimic the dermis and spatially assigned DPCs and KCs to such dermal analogs, creating an array of HF organoids by using 3D printing technology [44]. Kageyama *et al.* achieved the formation of HF grafts (HFGs) by encapsulating mouse embryonic MSCs or human DPCs along with epithelial cells in collagen microgels, facilitating EMIs within contracted collagen gels [45]. Similarly, keratin, a key component of the epidermis, has been used in the construction of the epidermis [103] and other skin appendages, such as DPC spheroids [61].

Polysaccharides such as HA are among the major components of the ECM that is widely distributed in the dermis [104]. Kalabusheva *et al.* demonstrated that HA promotes the proliferation of KCs and DPCs, leading to increased organoid size in cultures consisting of KCs and DPCs [105]. HA offers advantages such as a stable composition and fully controlled physical and chemical properties [92, 106]. However, it does not support cell attachment [107] and is often combined with protein-based materials to enhance cellular adhesion. Other non-skin-specific proteins or polysaccharides, such as silk proteins [63, 108], alginate [82], and chitosan [65], have also been used in the construction of HF or SG organoids in combination with other biomaterials.

To address the limitations of natural hydrogels, synthetic hydrogels have emerged as promising alternatives for organoid construction. These synthetic hydrogels have good biocompatibility and tunable mechanical properties, allowing for controlled differentiation and recapitulation of *in vitro* tissue morphogenesis [101]. GelMA is a gelatin-modified light-curable hydrogel that retains the Arg-Gly-Asp (RGD) sequence and temperature sensitivity of gelatin and has been widely used in cell culture, 3D bioprinting, and drug delivery because of its good biocompatibility, excellent molding properties, and tunable physicochemical properties [92, 106, 109]. GelMA has been reported to load MSCs, DPCs or SKP cells, and efficiently regenerate SGs or HFs *in vivo*. [110–112]

Polyethylene glycol (PEG) is another commonly used synthetic polymer in organoid cultures [101]. It is bioinert and is often functionalized with reactive end groups such as acrylates, thiols, and N-hydroxyl succinimide (NHS) esters to incorporate key ECM biofunctions [113–115]. There are increasing reports on the construction of organoids using PEG mixed with HA [116], adhesion peptides [117], and GelMA [118]. Although the utilization of these chemically defined hydrogels in the construction of skin-based organoids is relatively rare, there is ongoing research focused on developing organoids using these chemically defined hydrogels.

Each biomaterial has advantages and limitations. As summarized in Figure 5, natural materials closely resemble the ECM of original tissues, resulting in excellent biocompatibility and biological activity, which promote cell proliferation and migration. However, natural materials exhibit complex and variable compositions, and their physical or chemical properties are not stable or controllable. In contrast, synthetic materials are highly processable, easily modifiable, and can be prepared under well-defined and controlled conditions [95]. However, hydrogels prepared from synthetic materials are bioinert, which inevitably affects cell adhesion and growth. There is a growing tendency to combine natural and synthetic materials in the fabrication of ECMs for organoid culture, aiming to leverage the advantages of both while mitigating their respective drawbacks.

#### Critical signaling molecules

Bioengineering techniques and 3D scaffolds help stem cells self-organize or distribute in a controlled manner; nevertheless, they are insufficient to direct cells to commit oriented differentiation and produce functional organoids. Therefore, the modulation of signaling molecules in the culture system to mimic *in vivo* skin morphogenesis is another critical determinant for the generation of organoid models.

**Figure 6:**
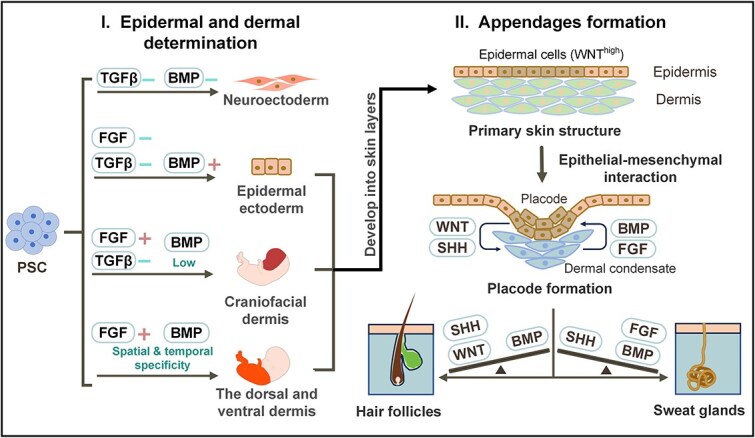
The signaling pathways involved in skin cell differentiation and appendage formation. The inhibition of the BMP and TGF-β signaling pathways (dSMADi) promotes the neural fate of PSCs. BMPs are generally accepted as epidermal inducers. BMP activation promotes epidermal fate when WNT is present, as WNT inhibits FGF signaling to prevent neural differentiation. NC cells are the main contributors to the development of the craniofacial dermis. BMP and FGF signaling are essential for NC induction, and the levels of these signaling molecules determine the cell fate. Low BMP expression and moderate FGF activity induce early induction of NC, and high levels of BMP and FGF promote epidermal and neural fates, respectively. The dorsal and ventral dermis have different developmental origins. The BMP and FGF signaling pathways are involved in the spatially and temporally specific development of the dermis. For example, BMP functions as an inducer of lateral mesoderm (contributing to the ventral dermis) specification, and BMP inhibition is required for paraxial mesoderm (contributing to the dorsal dermis) formation and differentiation. After the primary skin structure is established, EMI is critical for the formation of various skin appendages. During epithelial development, dispersed cells in the epidermal layer respond to elevated WNT signaling (WNT^high^) and aggregate to form placodes. These placodes subsequently SHH, which recruits adjacent mesenchymal cells to form DCs and produces BMP inhibitors. Repeated EMI drives the formation of diverse HF cell lineages and the maturation of HF structures. DC signals are temporally regulated during skin morphogenesis. If DCs produce BMP and FGF signals that suppress epithelial-derived SHH production, the placode will develop into SGs

The interplay of the fibroblast growth factor (FGF), bone morphogenetic protein (BMP), WNT, and transforming growth factor beta (TGF-β) pathways plays a crucial role in the development of the skin epidermis and dermis [13, 32, 119–121], and the precise regulation of one or more of these pathways enables the production of specific skin cells or skin organoids from PSCs (Figure 6) [48, 56, 57, 67, 94].

BMP4 is a generally accepted epidermal inducer. In chick embryos, BMP signaling directs epidermal fate in the presence of WNT; conversely, a lack of exposure to BMP and WNT permits FGF to induce a neural fate [122, 123]. By regulating FGF, BMP, WNT, and TGF-β signaling activity, all major ectodermal lineages, including surface ectoderm and NC cells, have been induced from human PSCs [32]. For example, high levels of BMP promote epidermal induction, and reduced BMP signaling favors the early induction of the NC cells [32, 124]. In Lee’s skin organoid model, treatment of embryoid bodies with BMP4 induced surface ectoderm, giving rise to the epidermis, and a subsequent morphogen cocktail with BMP inhibition and bFGF activation promoted the appearance of NC cells, giving rise to the craniofacial dermis [48]. Compared with the well-known surface ectodermal origin of the epidermis, the dermis develops from various germ layers according to its anatomical location. The understanding of dermal development remains limited, despite previous studies indicating the involvement of the TGF-β, BMP, and FGF pathways in the development of the dermis from the mesoderm [125]. To date, dermal organoids derived from the mesoderm have rarely been reported.

After the basic epidermal and dermal layers have developed, epithelial–mesenchymal cross-talk is critical for the generation and patterning of skin appendages, including HFs and SGs [13, 126]. During epithelial development, dispersed epidermal cells react to elevated WNT signaling and aggregate to form placodes [127, 128]. Placodes release secrete sonic hedgehog (SHH) signals to recruit adjacent mesenchymal cells, leading to the formation of DCs, which are precursors to DPCs. If the underlying DCs produce signals of BMP inhibitors, e.g. Noggin, the placodes will form HFs [128, 129]. When the underlying mesenchyme produces robust BMP signaling, the placodes differentiate into SGs [52, 126].

Researchers have employed a variety of genetic or chemical techniques to manipulate these pathways to regenerate skin appendages or establish corresponding organoids. WNT signaling plays a decisive role in the generation of HFs. Subcutaneous injection of WNT into the bald scalp can reactivate hair growth [130], and pretreatment of collagen-encapsulated iPSCs with WNT10b promoted the generation of bioengineered HFs [67]. *Lymphoid enhancer-binding factor 1 (Lef1)*, a downstream transcription factor of the WNT pathway [131], markedly augmented the hair-inducing capability of engineered HFs when introduced into DPCs [44]. Moreover, other molecules, including CHIR99021 (a GSK3β inhibitor that promotes canonical WNT activation) [44, 50, 94], purmorphamine (SHH activator) [94], and Noggin (competitive BMP inhibitor) [58], have been shown to promote HF generation. Conversely, XAV-939, an inhibitor of WNT [132], inhibits the formation of HF-like structures and subsequent HF budding when added to the hair germ [43].

In contrast, the development of SGs primarily depends on factors such as ectodysplasin A (EDA), BMPs, and FGFs. These factors inhibit SHH signals within the epithelial region, promoting increased BMP signaling activity and facilitating the formation of SGs [120, 126, 133]. Both the WNT and EDA pathways seem to participate in promoting placode formation and enhancing SG formation [26, 120, 134]. Moreover, EDA has been reported to direct KC conversion into SG cells [40, 56, 134].

In addition to molecules that regulate fate determination, other substances have also been used to optimize skin organoid culture. Inhibitors of the TGF-β signaling pathway, such as A83-01 or SB431542, increased the colony formation efficiency of iPSCs and were essential for epidermal induction [40, 50, 68, 135]. Activators of the cyclic adenosine monophosphate (cAMP) pathway, e.g. forskolin, are often used to promote cell proliferation in culture [10, 11]. Additionally, selective inhibitors of Rho-associated kinases, such as Y27632, reduce stem cell apoptosis and modulate the spatial distribution of epithelial and mesenchymal cells, thereby increasing the rate of HF budding [136].

### Various types of organoid models

Through the integration of stem cells, bioengineering methods, 3D scaffolds, and signaling molecules, a range of skin models that closely mimic the structure and function of the skin have been developed. A summary of the preparation methods and applications of these organoid models of skin is provided in Table 1.

**Table 1:** Overview of organoid models of skin

Organoid identity	Species	Cell source	Biomaterials	Bioengineering methods	Application
**Epidermal organoids**	*Rodent*	Epidermal KCs	Matrigel	Spherical aggregates	Organoids expand over 6 months, maintaining the structure of the epidermis [10]. Modeling of skin infections caused by *Staphylococcus aureus* [12].
	*Human*	Primary skin epidermal cells	Matrigel	Spherical aggregates	Contributing to epidermal regeneration at wound sites [55]. Modeling of dermatophyte infections caused by *T. rubrum* [11].
**HFs (dermal cells)**	*Rodent*	DPCs and ASCs	Chitosan	Sequential assembly of DPCs and ASCs to develop into core-shell spheres	Reconstruct cellular arrangements and microenvironmental niches for hair formation [59].
		DPCs	Gelatin/alginate [60], keratin [61]	Layer-by-layer self-assembly [60], spherical aggregates [61]	New HF regeneration following DP spheroid implantation [60, 61].
	*Human*	hDPCs	NA [62, 137], silk-gelatin hydrogel [63].	Hanging drop [62], spherical aggregates [63, 137]	DP aggregates induce HF neogenesis *in vivo* [62, 137]. Modeling for drug screening of androgenic alopecia [63].
		hDPCs, matrix cells, and DS cup cells	Matrigel	Spherical aggregates	A microengineered system for scalable production of hDPC spheroids and HF regeneration [58].
**HFs (containing epidermal and dermal cells)**	*Rodent*	Embryonic epithelial and mesenchymal cells	Microwell-array PDMS chip [45], Matrigel [43]	Self-assembly [43, 45], large-scale production on the chip [45]	Establishment of *in vitro* HF model [43, 45], enabling the large-scale production of HF germs on PDMS chip. [45]
		MSCs and epidermal cells	GelMA cores and HA shells	Microfluidic-assisted technology	One-step generation of biomimetic microspheres for hair regeneration [66].
	*Rodent/* *Human*	Mouse epidermal cells, human DP cells [83, 138] or HUVECs [138]	Collagen type I, [83, 138] microwell-array PDMS chip [138]	Self-assembly in contracted collagen gels, large-scale production using automated spotter	Efficient generation of HF upon transplantation [83, 138]. HF containing HUVEC produced higher hair inductivity [138].
	*Human*	Fetal/adult epithelial and mesenchymal cells	Matrigel or collagen	Self-assembly into spherical aggregates	*In vitro* models of human HF-like structure, which are valuable for drug screening [139].
		Primary or N/TERT-1 KCs, hDPCs and fibroblasts	Collagen	3D installation	Generation and integration of HF-primed spheroids in bioengineered skin constructs [46].
**SeG**	*Rodent*	Sorted Blimp1+ cells from mouse epidermis	Matrigel	Spherical aggregates	Models for the mechanical exploration of acne vulgaris [39].
**SG**	*Rodent*	Epithelial progenitors [82], MSCs [64]	Gelatin/alginate scaffold with dECM of plantar dermis	Cells were 3D bioprinted with matrix biomaterials	SG–like matrix contributed to the conversion of epithelial progenitors [82] or MSC [64] into functional SGs and promoted SG recovery *in vivo*.
		SG cells from adult paw pads	Matrigel	Spherical aggregates (ULA)	Regeneration of epidermis and SG when SG organoids were transplanted *in vivo* [40].
		MSCs	Collagen/chitosan porous scaffold	Scaffold loaded with Lipofectamine 2000/pDNA-EGF	Accelerate wound healing process and induce regeneration with SG-like structures [65].
	*Human*	Epithelial cells from eccrine SG	Matrigel	Spherical aggregates(ULA)	Models of human eccrine SG [140].
		Primary SG cells	NA	Spherical aggregates (hanging drop)	Models of human eccrine SG in response to the stimulation [41].
		Forced expression of ectodysplasin-A in KCs	Matrigel	Conversion of KC into SG cells, assisted with microfluidic technology	SG organoids exhibit features of native SGs and develop into fully functioning SGs after transplantation into mouse models [56].
**Skin organoids**	*Rodent*	ESC or iPSCs	Matrigel	Spherical aggregates (ULA)	*In vitro* skin organoids containing epidermis and dermis, as well as HF. [50]
	*Human*	Endothelial cells, fibroblasts and KCs	NA	Spherical aggregates (ULA)	Organoids exhibited specific structure with surface-anchored KCs enveloping a central stromal core [57]
		hESCs and hiPSCs	Matrigel	Spherical aggregates	Hair-bearing human skin models generated entirely from PSCs [48, 49]
		hiPSCs	Matrigel, Collagen I	Self-assembly, followed by ALI	Modeling atopic dermatitis by bacterial colonization and infection [47].
		hiPSCs	Matrigel	Spherical aggregates(ULA)	Skin organoid models of SARS-CoV-2 infection [69] and EB [141].
**Skin-on-a-chip**	*Human*	HaCaT cells, HS27 fibroblasts, HUVECs	PET membranes coated with fibronectin, PDMS channel	Microfluidic-assisted technology	Modeling of skin inflammation and edema; evaluating the efficacy of therapeutic drug [87].
		Fibroblasts and hKCs	PDMS chips	ALI and Microfluidic-assisted technology	A wrinkled skin-on-a-chip model was developed using cyclic stretching. This model can be used to study skin aging and evaluate anti-wrinkle cosmetics and medications [88].
		HaCaT and leukemic lymphoma cell line (U937)	Assembly of PMMA, PS and PDMS sheet.	Microfluidic-assisted technology	Development of an immune competent *in vitro* model of human skin. This model has been used to evaluate drug efficiency and toxicity [89].

Abbreviations: not applicable (NA), ectodysplasin-A (EDA), gelatin methacryloyl (GelMA), human adult keratinocyte cell line (HaCaT), poly(methyl methacrylate) (PMMA), polystyrene (PS), polydimethylsiloxane (PDMS), polyethylene terephthalate (PET), ultralow attachment (ULA)

#### Epidermal organoids

Although 2D murine/human EpSC cultures were established long ago [73], a feeder- and serum-free murine epidermal organoid culture system was only recently established, enabling the long-term expansion (>6 months) of adult EpSCs [10]. The culture medium combines high calcium concentrations; the activation of cAMP, FGF, and R-spondin signaling; and the inhibition of BMP signaling. The epidermal organoids exhibited layered structures; the dividing epithelial cells expressing proliferation markers were located in the outermost layer, and the differentiated KCs expressing Keratin 6A, Keratin 1 and Loricrin formed toward the center of the organoids. A similar epidermal organoid culture system derived from human skin was also established by using chemically defined medium containing activators of cAMP, epidermal growth factor (EGF), and WNT3a and inhibitors of the TGF-β pathway. These human epidermal organoids replicate the morphological, molecular, and functional characteristics of the human epidermis and maintain their viability for up to 6 weeks [11]. The discrepancy in *in vitro* expansion time between human and murine epidermal organoid systems might be attributed to species differences, and further optimization of the culture system of human epidermal organoids remains challenging.

#### Organoids or aggregates mimicking skin appendages

Skin appendages are highly complex structures. Dynamic interactions of various chemical signals between the epidermis and dermis are necessary to promote the induction and maturation of skin appendages during fetal development [13, 142]. Consequently, it is challenging to create organoids that mimic skin appendages solely through the self-assembly of a single type of stem cell. Instead, a common approach involves the assembly of cells from different skin layers in specific spatial arrangements within a bioengineered scaffold. Various terms, such as organoids, aggregates, and 3D spheroids, have been utilized to describe the 3D multicellular structures that replicate the distinctive structure and function of different skin appendages.

##### Human follicle organoids

The HF consists of cylindrically multilayered KCs [including HF stem cells (HFSCs)] and mesenchymal hair-inductive DPCs located at its base [13, 84]. DPCs play crucial roles in generating dermal cell populations, including dermal sheath (DS) cells, which are located within the connective tissue sheath surrounding the HF [143, 144]. HFs not only contribute to hair growth but also play a crucial role in skin regeneration after injury [145–147]. Therefore, numerous attempts have been made to regenerate HFs both *in vivo* and *in vitro* [43, 58, 79, 139, 148–150].

Transplantation experiments conducted in nude mice have demonstrated that the crosstalk between HFSCs and DPCs is responsible for HF generation [84, 151–153]. Furthermore, DPCs play an essential role in the hair-inducing process, but HFSCs can be replaced by other epidermal cells [44, 46, 94]. However, human DPCs rapidly lose their hair-inductive potential in conventional culture [79, 154]. To overcome this limitation, 3D DPC cultures were established and showed improved efficacy in HF neogenesis [38, 60, 61, 63, 137]. By imitating the *in vivo* microenvironment of DPCs, coculturing DPCs with hair matrix cells [58], DS cells [58], KC cells [44, 46, 57], or adipose-derived stem cells (ASCs) [59] can enhance hair regeneration and HF maturation. Additionally, various bioengineering methods have been employed to assemble these cells within biomaterials, aiming to mimic the specific structure of HFs. Specifically, Abaci *et al.* induced hair generation by implanting aggregates of DPC and KC mixtures into a 3D-printed dermis composed of fibroblasts and type I collagen gel. The encapsulation of human umbilical vein endothelial cells (HUVECs) in the dermis promoted capillary formation and allowed efficient hair growth [44]. Using the 3D bioprinting technique, Kageyama successfully prepared hair microgels by incorporating DPCs and KCs in collagen gels [83]. Upon implantation into the back skin of murine models, these engineered microgels efficiently induced HF and shaft regeneration. In a recent study conducted by Huang *et al.*, unsorted MSCs and epidermal cells from newborn mice were encapsulated in GelMA and photocurable catechol-grafted materials, respectively, and these core–shell biomimetic microspheres significantly enhanced hair regeneration [66]. Notably, however, all the above DPC or 3D cell cultures have to be transplanted into animal models to exert HF-inducing effects.

In recent years, significant progress has been made in generating hair *in vitro*. Kageyama *et al.* generated HFs in vitro by coculturing unsorted epithelial cells and mesenchymal cells from the skin of mouse embryos in Matrigel. This EMIs produced cell aggregates with mesenchymal cells in the outer layer and epithelial cells in the inner layer, leading to nearly 100% efficiency of HF generation in each aggregate [43]. Later, the same research group demonstrated that *in vitro* spherical aggregation and subsequent hair PEG-like sprouting can also be achieved by coculturing human fetal/adult epithelial and mesenchymal cells in Matrigel or collagen I [139]. A common feature observed in these studies is the involvement of dermal DPCs (or unsorted mesenchymal cells) and epithelial components in the formation of HFs, further indicating that EMI is essential for the artificial generation of HFs [84]. Notably, the hair-inducing capacity of mesenchymal cells may decline with age. In an epithelial–mesenchymal coculture model [155], mesenchymal cells derived from neonatal mice generated significantly more HFs than those from older (>2 months) mice.

Alternative approaches have also been developed to induce HF generation without relying solely on DPCs. For example, SKPs, a subset of specialized dermal cells, also show hair-inducing properties. SKPs originate from the NC and are multipotent stem cells that form self-renewing spheroids *in vitro* and can differentiate into various cell lineages [156, 157]. The combined transplantation of cultured EpSCs and SKPs successfully reconstituted functional HFs in nude mice [158]. In addition, DPCs can be reprogrammed from human dermal fibroblasts [159] or iPSC-derived mesenchymal cells [94].

##### Sebaceous gland organoids

The SeG is another important epidermal appendage that originates mainly from an HF [25] and forms the pilosebaceous unit together with the HF [90]. It is a unilobular or multilobular structure that connects to the HF through a duct lined by epithelial cells. In addition to HF-associated SeGs, independent free SeGs exist at mucosal margins and areas of modified skin [25, 39, 160, 161]. Research on SeG organoids is relatively rare, probably because of their close association with mature HFs [162]. Feldman *et al.* demonstrated that individual mouse Blimp1+ cells possess the capacity to form SeG organoids *in vitro*. These organoids express known SeG markers and display a lipidomic profile comparable to that of native SeGs *in vivo* [39].

##### Sweat gland organoids

The SG consists of a secretory coil, comprising myoepithelial, secretory clear (serous) and dark (mucous) cells, and a connecting duct that opens directly onto the skin surface [163]. The isolated SG cells rapidly differentiate into KCs and lose their specific phenotypic characteristics in 2D culture; [40, 164, 165] therefore, various methods, such as hanging drop cultivation or Matrigel encapsulation, have been used to construct 3D SG cultures. Klara *et al.* reported that human SG cells grown via the hanging drop cultivation method retain most of their markers and respond to cholinergic stimulation [41]. In a murine SG 3D culture, epithelial cells isolated from SGs were embedded in Matrigel and cultured in medium supplemented with EGF, basic FGF (bFGF), EDA, A83–01, forskolin, and BMP4, leading to the formation of SG organoids. These organoids retained significant stem cell properties and exhibited the ability to differentiate into either SG cells or epidermal cells [40].

SGs are very tiny and scattered throughout the dermis layer, which makes them difficult to acquire. Other cell sources, such as EpSCs, KCs, MSCs, ESCs, and amniotic fluid-derived stem cells, have demonstrated the potential to transdifferentiate into SG cells by genetic or chemical manipulation [64, 166, 167]. Considering the enormous impact of the niche on cell fate, 3D printing techniques have also been used to provide a biomimetic microenvironment to drive SG differentiation. For example, MSCs were bioprinted into a specific matrix from the mouse plantar region dermis and directed toward fate conversion into functional SG cells, leading to functional regeneration of SGs in burn mouse models [64]. Moreover, by culturing human epidermal KCs that overexpress EDA in specialized SG culture medium, KCs were efficiently converted into induced SG cells. These cells were further embedded in Matrigel and developed into SG organoids that closely mimicked the structural and biological features of native SGs. These organoids were successfully passaged and maintained for up to 3 months, and they demonstrated the ability to differentiate into functional SGs following *in vivo* transplantation [56]. This research underscores the significant translational potential of SG regeneration in the clinic.

#### From pluripotent stem cells to skin organoids

As described previously, the skin is a complex, stratified organ that harbors multiple appendages. Constructing appendage-bearing skin in culture is a great biomedical challenge, and skin organoids that nearly mimic complete skin have only recently been established entirely from PSCs. By modulating the TGF, FGF, and BMP pathways, Lee *et al.* produced mouse skin organoids from ESCs and iPSCs during an incubation period of 20–30 days. Mouse PSCs were treated with SB4315432 and BMP in 3D culture to induce surface ectoderm, followed by treatment with FGF and LDN-193189 to induce placodal epithelium. Next, the aggregates were cultured into skin organoids containing self-organized skin layers and skin appendages, including HFs, SeGs and adipocytes. [50] Using a similar induction system, Lee *et al.* prepared human skin organoids that mimic the complex skin structure from human iPSCs. By sequentially modulating the TGF-β and FGF signaling pathways to co-induce nonneural ectoderm and NC cells within cell aggregates, cyst-like skin organoids consisting of stratified epidermis, fat-rich dermis and pigmented HFs with SeGs were induced within a 4–5-month incubation period. Notably, these organoids formed a sensory neuron and Schwann cell network, which in turn formed nerve-like bundles that targeted Merkel cells within HFs [48, 49]. Despite significant advances in the construction of human skin organoids, several challenges still persist. For example, there is a lack of SG formation [48, 49] and an absence of type VII collagen in the structure of epidermal–dermal junctions (EDJ) in skin organoids [141], and such limitations should be considered when modeling certain diseases. Moreover, off-target differentiation of hyaline cartilage in the tail region of skin organoids can be observed [48, 49], although this has reportedly been improved by additional stimulation via the WNT signaling pathway during the induction period. WNT activation also increased the size of human skin organoids, and such organoids could be further developed into skin analogs via the ALI culture method [47].

### The application of organoid models in biomedical research

Traditional ALI skin models have been widely introduced in disease modeling and drug tests, such as the study of bacterial infections, wound healing, inflammatory cutaneous diseases, screening of antimicrobial peptides, and evaluation of cosmetics [9, 168–170]. Based on ALI models, microfluidic skin-on-a-chip platforms, such as cocultures of monocytes with KC-based epidermis in a dual-channel microfluidic setting [89], have been introduced to closely mimic natural human skin and help determine drug efficiency and toxicity.

**Figure 7:**
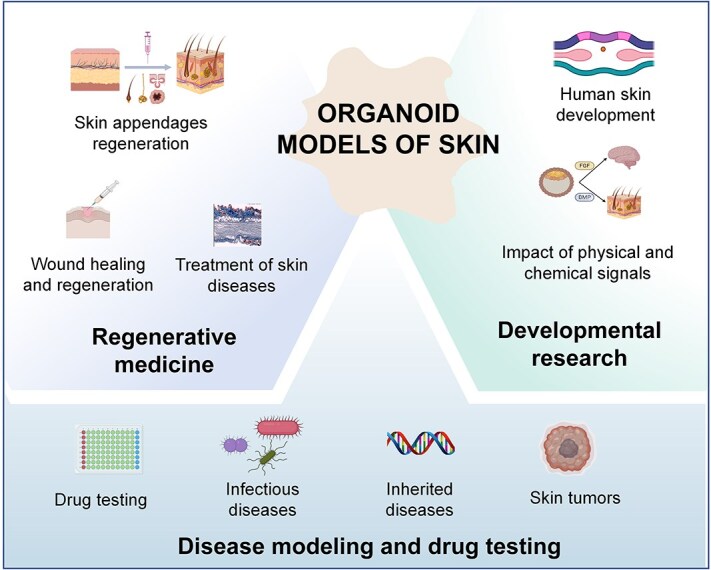
Biomedical applications of organoid models of skin. Skin-based organoids have a wide range of applications, encompassing disease modeling, regenerative medicine, and developmental research. These organoids serve as invaluable tools for mimicking and studying various skin diseases in a controlled *in vitro* setting. In addition, they offer a platform for screening potential drugs and cosmetics for their efficacy and safety. From a regenerative medicine perspective, organoid models of skin hold promise for tissue engineering and transplantation purposes. Furthermore, these organoids provide a platform for investigating the complex processes involved in human skin development and provide a better understanding of embryonic and postnatal skin development. Created with BioRender.com

The generation of organoid models of skin, especially skin appendage-bearing organoids, has offered novel opportunities for skin disease modeling, regenerative medicine, and developmental research (Figure 7). These organoids demonstrate structural, transcriptomic, and proteomic similarity to their tissue of origin [10, 11, 48, 49, 57], highlighting their exceptional potential in many aspects of skin research.

#### Disease modeling

Epidermal organoids present stratified histological and morphological characteristics of the epidermis. Xie *et al.* conducted a study using mouse primary epidermal organoids to investigate their susceptibility to methicillin-resistant *Staphylococcus aureus* (MRSA) USA300 infection and to explore the mechanisms of antimicrobial drug action [12]. The findings revealed that epidermal organoids support the colonization and invasion of MRSA USA300, as evidenced by the presence of swollen epithelial cells with nuclear necrosis and the secretion of inflammatory factors. Similarly, Wang *et al.* established human epidermal organoids to investigate the phenotype and underlying mechanism of dermatophyte infections caused by *Trichophyton rubrum* [11]. *T. rubrum* infections of human epidermal organoids accurately reflect many aspects of known clinical pathological conditions. Additionally, the repression of IL-1 signaling may contribute to the development of chronic infections with *T. rubrum* in human skin.

hiPSC-derived skin organoids recapitulate the complexity and function of full-thickness human skin. Jung used such a model to study atopic dermatitis caused by *S. aureus* (SA) colonization and infection. The findings revealed disruption of the skin barrier and upregulation of inflammatory cytokines originating from both the epidermis and dermis. Furthermore, the therapeutic effects of pretreatment with *Cutibacterium acnes* on SA-infected models were investigated [47]. Ma *et al.* studied the ability of severe acute respiratory syndrome coronavirus 2 (SARS-CoV-2) to infect hiPSC-derived skin organoids containing HFs and neural cells. They reported that Keratin 17+ HFs and different types of neural cells can be infected by SARS-CoV-2, providing evidence for the association between SARS-CoV-2 infection and hair loss [69].

Skin organoids are also promising tools for studying inherited diseases and skin tumors. For example, human-induced PSCs (hiPSC)-derived hair-bearing skin organoids represent a novel approach for modeling diseases such as epidermolysis bullosa (EB). This inherited disease is characterized by abnormalities in the expression or structure of components in the EDJ. In organoids, basal KCs attach to the basement membrane through EDJ-like structures, although the lack of type VII collagen needs to be addressed [141]. Similar to other types of organoids, the utilization of patient-derived or gene-edited donor hiPSCs allows the simulation of a wide range of inherited skin diseases and facilitates drug screening experiments. Additionally, recent studies have utilized organ models to explore the development, progression, and therapy resistance of skin cancer, such as melanoma, providing valuable insights into this type of skin cancer [171].

#### Regenerative medicine

Skin-based organoids may serve as viable cell sources for reconstructing damaged skin and enhancing wound healing efficiency. The transplantation of epidermal organoids [55], HF organoids [42, 60, 172], SG organoids [40, 56], and iPSC-derived skin organoids [48, 67] into wound models has been shown to promote skin regeneration and the reconstruction of corresponding skin appendages. For example, human epidermal organoids were delivered to the sites of severe skin wounds with the aid of a micro-atomization device, where they facilitated skin reconstitution and wound healing [55].

Extensive evidence supports the crucial role of HFs in the processes of wound healing and skin remodeling. Clinical studies have demonstrated that the transplantation of autologous HFGs into chronic ulcers significantly accelerates wound healing [146, 173, 174]. However, the use of HF organoid technology is currently limited because of its complexity, small-scale production, and high cost. Consequently, HF organoid transplantation research has focused mainly on regenerating HFs in patients with alopecia. Several studies have successfully shown that HF organoid transplantation effectively regenerates HFs when they are transplanted into mouse models [43–45, 83]. This novel approach holds immense promise for the treatment of alopecia.

The restoration of SGs is a significant challenge for individuals with extensive skin defects. However, encouraging progress has been made in this field. By transplanting SG organoids derived from reprogrammed epidermal KCs or SG epithelial cells into a mouse wound model, researchers have successfully restored the crucial functions of SGs during wound healing [40, 56]. This advancement highlights the significant potential for SG regeneration in patients with large skin defects.

With respect to iPSC-derived skin organoids, the transplantation of these organoids into nude mice revealed remarkable integration of the organoid-derived epidermis with the host epidermis. Notably, hair growth was observed in ~55% of the xenografts. Additionally, the epidermis of the xenografts presented cornified layers and retro-ridge-like structures comparable to those observed in adult facial skin [48, 49]. With careful control of safety and reproducibility, these skin appendage-bearing organoids can contribute to the morphological and functional recovery of skin in patients with severe burns or wounds. Moreover, it is worth noting that iPSC-derived organoid models containing both epithelial and mesenchymal tissue significantly reduce the degree of skin fibrosis and increase the activity of EpSCs in patients with localized scleroderma [68]. This study highlights potential applications of organoids in addition to regeneration.

#### Developmental research

A precise understanding and translation of embryogenesis is the foundation of the *in vitro* generation of organoids. By leveraging insights into how embryonic development leads to organ formation, PSCs were differentiated to generate skin organoids with multiple lineages. Thus, the organoid technique has opened a new avenue for advancing our comprehension of skin development [175]. On the basis of scRNA-seq and immunostaining data, hiPSC-derived skin organoids (~150 days of culture) have been shown to represent human full-thickness skin tissues during the second-trimester stage of fetal development [48, 49]. Therefore, tracking and analyzing gene expression and signaling pathways during the formation of skin organoids would provide valuable insights into human skin development. Recently, Gopee *et al.* revealed that the crosstalk between nonimmune and immune cells is pivotal in human HF formation and vascular network remodeling via the use of skin organoids [176], thereby advancing our understanding of human skin morphogenesis. Manipulating the signaling pathways that govern skin development could also have a profound impact on the generation of skin organoids. For example, previous studies have shown that the activation of WNT signaling through the use of the GSK-3β inhibitor CHIR99021 can promote the growth of skin organoids and prevent off-target cartilage differentiation [47]. In addition, the skin organoid model is an excellent platform for studying skin innervation mechanisms, as it allows the incorporation of nerves into existing skin equivalent cultures [48, 49, 177].

## Conclusions

Organoid models of skin have emerged as an effective platform for deepening the understanding of skin development, exploring skin-based diseases, and identifying new strategies for skin function reconstruction. This review has provided a comprehensive overview of their construction strategies and recent advances, with a particular emphasis on the importance of appendage-bearing models, which offer research systems with greater physiological relevance. However, the journey toward creating idealized skin organoid models is far from complete. The field continues to grapple with universal challenges inherent to organoid science, including morphological and functional heterogeneity, batch-to-batch variability that complicates reproducibility, and a reliance on labor-intensive protocols that hinder scalability for high-throughput screening and clinical application. Beyond these general hurdles, skin organoids face specific limitations. For example, hPSC-derived skin organoids form a sphere-like cyst where hair shafts sprout inward, with hair bulbs protruding outward from the surface of the cysts. Moreover, the cornified epidermis accumulated in the core, which is opposite to the structure of normal human skin. Such unique inside-out structures could also be observed in epidermal organoids, HF organoids formed by epithelial-mesenchymal interaction etc. Additionally, skin organoids are still lack of some critical cell populations of normal human skin, such as SGs, blood vessels and immune cells. Consequently, future research may continue to focus on the following areas. Must be strategically directed toward several key frontiers. First, Achieving a higher fidelity of skin's complexity and functionality. Developing skin organoids that replicate the diverse cell types, specialized microenvironments, and intricate architecture of the skin is crucial. Additionally, these organoids should demonstrate the physiological functions of native skin, including barrier function, immune responses, and wound healing capabilities. Second, vascularization, innervation and integration of skin appendages are crucial. The incorporation of a functional vascular network is essential for nutrients and oxygen supply, moreover, nerve innervation and integration of skin appendages like HF, SGs, and SeGs into the organoids are crucial for developing comprehensive skin models that replicate natural skin features. Third, enhancing model stability, scalability, and reproducibility is essential for translational success. This will require a concerted push towards chemically defined and xeno-free culture conditions, as well as the adoption of cutting-edge bioengineering techniques and automated bio-fabrication to overcome current limitations. Through ingenious design and continued technological breakthroughs, the organoid platforms are poised to deliver transformative new strategies for disease modeling, drug discovery, and regenerative medicine.
